# Identification and Characterization of Volatile Organic Compounds Based on GC-IMS Technology in Different Organs of *Lilium brownii* var. *viridulum* and After Bud-Removal and Non-Bud-Removal Treatments

**DOI:** 10.3390/molecules30061238

**Published:** 2025-03-10

**Authors:** Xiaoling Li, Zhihui Wang, Chaowen Hou, Xiujuan Gong, Zexiang Liu, Yuhe Shi, Jianye Yan, Qiaozhen Tong

**Affiliations:** 1 School of Pharmacy, Hunan University of Chinese Medicine, Changsha 410208, China; jinli00@stu.hnucm.edu.cn (X.L.); 004595@hnucm.edu.cn (Z.W.); houcw@stu.hnucm.edu.cn (C.H.); gongxj@stu.hnucm.edu.cn (X.G.); liuzx@stu.hnucm.edu.cn (Z.L.); 20212112@stu.hnucm.edu.cn (Y.S.); 2 Science & Technology Innovation Center, Hunan University of Chinese Medicine, Changsha 410208, China; 3 Hunan Provincial Key Laboratory of Germplasm Resources and Standardized Cultivation of Bulk Genuine Medicinal Materials, Changsha 410208, China

**Keywords:** *Lilium brownii* var. *viridulum*, GC-IMS, fingerprint, multivariate analysis, volatile flavor properties

## Abstract

*Lilium brownii* var. *viridulum* (Longya lily) is a widely used medicinal and culinary plant in China that is valued for its potential applications and development opportunities. The bulbs of Longya lily contain a variety of active components; volatile oil, as one of the main biologically active compounds, has been widely studied, and the stems, leaves, and flowers of Longya lily are also rich in volatile organic compounds (VOCs) and related pharmacological effects, but the horizontal comparison of different organs of lily plants is lacking. In order to promote the sustainable development of resources, the composition characteristics and differences of bulbs, stems, leaves, and flowers, as well as two crop management methods (bud-removal and non-bud-removal), were comprehensively studied by GC-IMS technology in this study. Additionally, multivariate statistical analyses were used to identify the key components responsible for the observed differences among the plant organs and treatments. The research identified a total of 93 volatile organic compounds in Longya lily samples, primarily consisting of aldehydes, alcohols, ketones, and esters. If the VOCs of Longya lily are to be used as raw materials, it is advisable to choose flowers and leaves with a higher concentration of these components for harvesting. Notably, bulbs that were non-bud-removal exhibited a greater total content of volatile flavor substances compared to those that were treated with bud removal, with hexanal-D and (*E*)-2-hexenal-D being the most prevalent volatile organic compounds. This study provided theoretical support for the harvesting parts and crop management methods of Longya lily based on volatile organic compounds and promoted the high-quality development of the Longya lily industry.

## 1. Introduction

Lily refers to the dried fleshy bulbs of the plants *Lilium lancifolium* Thunb., *Lilium brownii F.E. Brown* var. *viridulum* Baker, or *Lilium pumilum* DC from the lily family. *Lilium brownii* var. *viridulum*, known locally as Longya lily, is one of the three main edible lilies that are cultivated in China and is often referred to as “southern ginseng” because of its tasty bulb, sticky texture, and high nutritional value. Longya lily is rich in various biologically active compounds, such as steroidal saponins, phenolic acids, polysaccharides, alkaloids, and volatile oils [1,2], which are commonly used in treating conditions like pulmonary tuberculosis, chronic obstructive pulmonary disease, depression, and diabetes [3,4,5]. Among these compounds, volatile oil is a key factor in assessing the quality of the lily. It is known for its expectorant, cough-suppressing, and asthma-relieving properties, showing significant effectiveness against chronic pneumonia and acute bronchitis [6]. Research has identified isopropyl palmitate in the volatile oil of *Lilium lancifolium* Thunb.; this substance is a low-viscosity, lipophilic, non-ionic surfactant that serves as a humectant, penetrant, and solvent for fragrances and colors. It also offers beneficial properties for skin care, including as a moisturizer and as a whitening agent, and has excellent water retention properties, making it suitable for use in cosmetics in substantial amounts [7]. Lily flowers are known for their strong fragrance and abundance of VOCs, which are often used as a premium raw material for producing perfumes and skincare products [8]. At present, research studies, both domestic and international, primarily concentrate on the volatile oil of Longya lily found in its bulbs and flowers [9,10,11]. In contrast, there is a lack of research examining the changes in aroma characteristics in its stems and leaves. Thus, studying the volatile organic compounds of Longya lily and comparing the similarities and differences among the volatile organic compounds from its stems, leaves, and flowers is a research topic that deserves attention.

Proper field management patterns are essential for stabilizing the yield and quality of lilies [12]. The timely removal of flower buds is a key crop management method for ensuring the centralized supply of nutrients for underground bulb growth and increasing lily yields [13]. There has been research conducted in China regarding increasing yield by removing lily flower buds. Luo Anhong revealed the significant effects of different flower removal periods and flower removal amounts on the yield of lily bulbs, and the results showed that the treatment of completely removing flower buds at the bud stage achieved the highest yield of 2385 kg/667 m^2^ [14]. Some studies have investigated and evaluated the effects of different crop management methods on the quality of Longya lilies; in general, the timely removal of flower buds can promote the flow of photosynthetic products to the underground bulbs, thus promoting the growth and expansion of the bulbs [15,16]. However, fewer reports exist on the effects of bud-removal and non-bud-removal treatments on volatile organic compounds in Longya lilies. To better understand the flavor differences in Longya lilies with different crop management methods, this study used Gas Chromatography–Ion Mobility Spectrometry (GC-IMS) to analyze the differences in the VOCs in Longya lilies with and without bud-removal treatments in order to clarify the types and contents of volatiles, to provide a theoretical basis for the crop management of Longya lilies, and for the development of the industry and resource sustainability of Longya lilies in the future.

Current methods for analyzing volatile organic compounds include gas chromatography–mass spectrometry (GC-MS), electronic nose (E-nose), and gas chromatography–olfactometry (GC-O), among other technologies. GC-IMS is a flavor detection technique that combines the excellent separating ability of gas chromatography with the high sensitivity of ion mobility spectrometry [17,18]. Because of its high separation and high sensitivity, it is now widely used in many fields, such as food quality control, the quality evaluation of traditional Chinese medicine, and origin identification [19,20,21]. In this study, the volatile organic compounds of four organs of Longya lily were identified, and their fingerprints were established after bud-removal and non-bud-removal treatments.

The volatile organic compounds of the four organs of Longya lily were analyzed using principal component analysis (PCA), clustering heat map analysis, and orthogonal partial least squares discriminant analysis (OPLS-DA), and the effects of bud-removal and non-bud-removal treatments on the volatile component content of Longya lilies were compared. This is an important theoretical reference for the in-depth study of Lilium and its further development and application in medicine, food, and other industries.

## 2. Results and Discussion

### 2.1. GC-IMS Spectroscopic Analysis of Longya Lily

**Figure 1:**
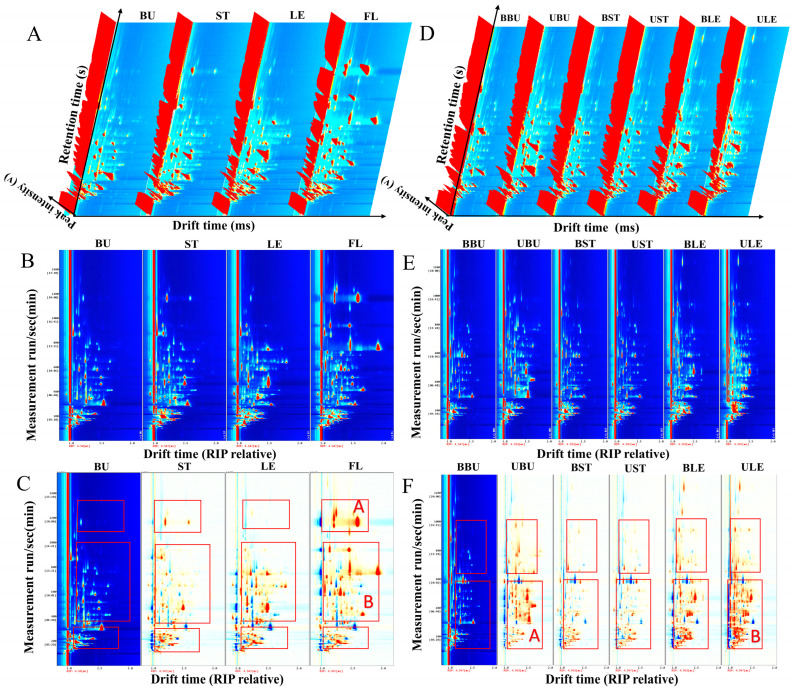
Three-dimensional morphology map (**A**); two-dimensional morphology map (**B**); and two-dimensional (comparative difference) fingerprint spectrum (**C**) of VOCs in different organs of Longya lilies. Three-dimensional morphology map (**D**); two-dimensional morphology map (**E**); and two-dimensional (comparative difference) fingerprint spectrum (**F**) of VOCs in Longya lilies with bud-removal and non-bud-removal treatments.

Figure 1A,D show the three-dimensional spectra of GC-IMS, with the three axes indicating the drift time (*X*-axis), retention time (*Y*-axis), and peak intensity (*Z*-axis). It can be seen from the graphs that the types of VOCs in the four organs of Longya lily, as well as their bud-removal and non-bud-removal treatments, are very similar, but the signal intensities are different, and the strength of FL is relatively high compared to the other three organs. As shown in Figure 1B,E, most of the signals in the Longya lily samples appeared in the range of 200–1200 s retention time, with a drift time between 1.0 and 2.0 s, which also suggests that the compositions of the VOCs in different Longya lily samples are similar.

To further visually compare the differences in the volatile organic compounds of different organs of the Longya lily and between the bud-removal and non-bud-removal treatments, the spectra of the BU and BBU samples were selected as references. The spectra of other samples were subtracted from the reference to obtain differential comparison charts for the different samples (Figure 1C,F). If the content of VOCs in the target sample is the same as that in the reference, the subtracted background will be white, while red indicates that the concentration of that substance in the target sample is higher than in the reference, and blue indicates that the concentration of that substance in the target sample is lower than in the reference [22]. As shown in the figure, the VOCs in the Longya lily flowers are significantly different from those in the bulb, stem, and leaf samples (Figure 1C), and the VOCs in the bulbs and leaves under the non-bud-removal treatment are significantly different from those under the bud-removal treatment (Figure 1F). The significant red areas at points A and B in the figure indicate that the compound content at points A and B is significantly higher than in other samples.

### 2.2. Identification of Volatile Organic Compounds in Longya Lily

The qualitative analysis of GC-IMS is based on the retention index (RI) of gas chromatography and the relative migration time of IMS. The IMS database was established by Shandong Haineng Scientific Instrument Co., Ltd. (Shangdong, China) through standard products. The results showed that a total of 124 signal peaks corresponding to 93 volatile organic compounds were identified in 10 samples, and the breakdown is shown in Table 1. In total, 33 aldehydes, 20 alcohols, 18 ketones, 10 esters, 4 heterocyclic, 4 hydrocarbons, 4 acids—including monomers (M) and dimers (D)—and unidentified volatile organic compounds are represented by numerical codes. The peak volume of volatile chemicals is calculated, the concentration of chemical substances is calculated by the internal standard method, and the quantitative quantity of chemical substances in different samples is realized.

### 2.3. Fingerprint Mapping Study

#### 2.3.1. Fingerprinting Study of VOCs in Different Organs of Longya Lilies

GC-IMS was used to characterize VOCs in the bulbs, stems, leaves, and flowers of Longya lilies to understand the changing patterns of VOCs in different organs. The original data in GC-IMS spectra were collated and compared with the built-in NIST and IMS databases to obtain the fingerprint of VOCs in the sample. The results showed that a total of 106 volatile chemicals (monomers and dimers) were detected in the four organs of Longya lily, including 31 aldehydes, 19 alcohols, 18 ketones, 7 esters, 4 acids, 4 hydrocarbons, 3 heterocyclic compounds, and 20 unknown compounds.

**Figure 2:**
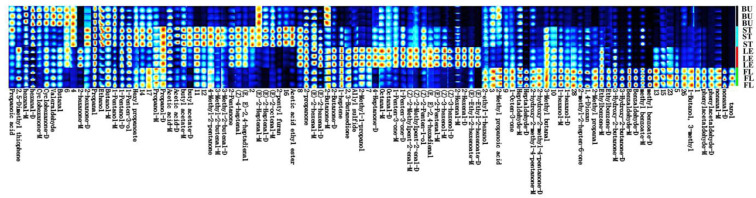
Fingerprints of volatile organic compounds in four organs.

Fingerprints were generated using the Gallery Plot plug-in, and the results are shown in Figure 2. The depth of color on the fingerprint can intuitively show the difference in the amount of VOCs in different organs. The relative concentrations of (E)-2-Heptenal-M (spicy, green vegetables, fresh) and 2-propanone (fresh, apple, pear) in BU samples were higher. Propanol-D (alcohol, pungent) was identified as the compound with the highest relative concentration in ST samples. The high content of LE samples included Octanal-M (aldehyde, waxy, citrus, orange, fruity, fatty); The FL sample contains relatively high levels of 2-Methyl propanoic acid (yogurt, rancid cream), 3-Methyl butanal (chocolate, fat), and nonanal-M (rose, citrus, strong oily).

#### 2.3.2. Fingerprinting of VOCs in Longya Lilies Undergoing Bud-Removal and Non-Bud-Removal Treatments

**Figure 3:**
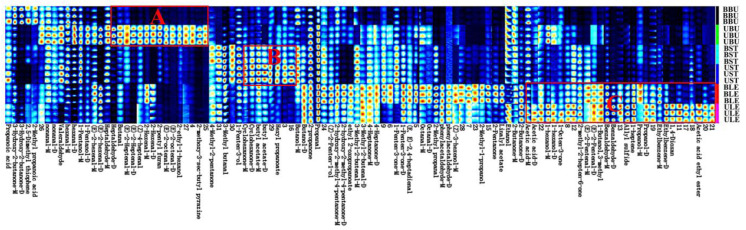
Fingerprints of volatile organic compounds in bud-removal and non-bud-removal treatments. Compounds in regions A, B and C in the figure are the main compounds that cause sample flavor differences, and the color of the signal peak ranges from blue to red, indicating that the concentration level of the compound goes from low to high.

A total of 108 volatile organic compounds (including monomers and dimers) were detected in the bud-removal and non-bud-removal treatments of Longya lily, including 30 aldehydes, 18 alcohols, 15 ketones, 6 esters, 4 acids, 4 hydrocarbons, 4 heterocyclics, and 27 unknown compounds. As can be seen from Figure 3, the difference in volatile organic compounds among the samples was obvious. The contents of 15 compounds, such as Heptaldehyde-D, Butanol-M, and (*E*)-2-Heptenal-M, in UBUs were higher than those in BBUs (Figure 3 region A). The compounds in region B mainly include Cyclohexanone-M, Cyclohexanone-D, and butyl acetate-M. The contents of these compounds in UST are relatively high, but the differences are not significant. The content of 29 compounds in ULEs in the C region is relatively higher than that in BLEs, which is consistent with the results of spectral analysis.

### 2.4. Multivariate Statistical Analysis

#### 2.4.1. Principal Component Analysis and Correlation Study of VOCs in Different Organs of Longya Lily

**Figure 4:**
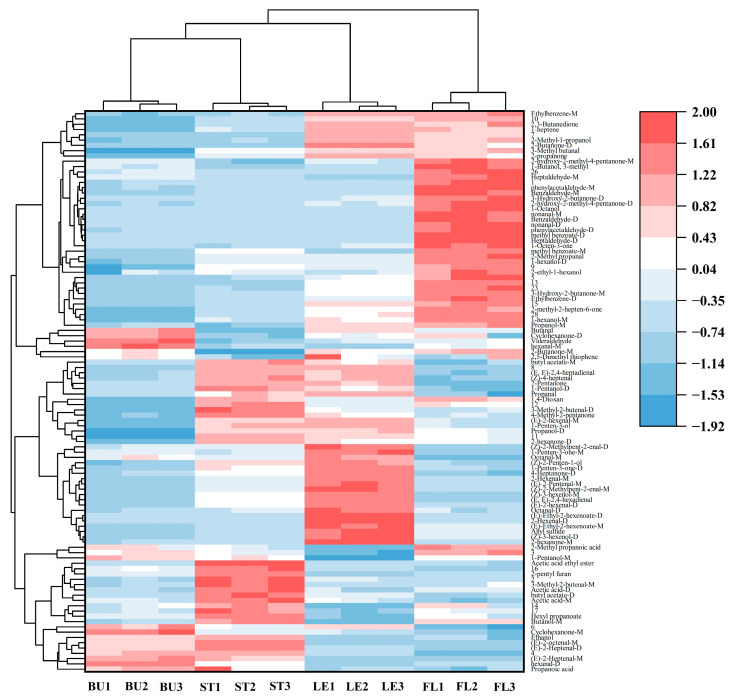
Heatmap of cluster analysis of different organs of Longya lilies.

Heatmap clustering analysis was performed using Origin 2022 to better understand the differences between samples from different organs of Longya lily, and clustered heatmaps of the samples in different regions were generated using the peak areas of the 106 fractions found as variables (Figure 4). The lightness and darkness of the signal peak colors in the graph represented the concentration of the substances, with blue representing a low expression negative correlation and red representing a high expression positive correlation [23]. Preliminary comparative analysis of the fingerprints shows that there are both common peak areas and characteristic peaks of VOCs in different organs of Longya lily, indicating that there are both similarities and differences between the composition and content of Longya lily flavors in different organs and that this difference is an important basis for distinguishing between the identification models of different organs of Longya lily. The major VOCs in BU were mainly aldehydes and alcohols, such as butanol-M, butanal, valeraldehyde, hexanal-M; the major VOCs in ST were mainly aldehydes and esters, such as 3-methyl-2-butenal-D, acetic acid ethyl ester, 3-,ethyl-2-butenal-M, butyl acetate-D, butyl acetate-M, and hexyl propanoate; the main VOCs in LE were mainly aldehydes and alcohols, such as (Z)-2-methylpent-2-enal-D, octanal-M, (*Z*)-2-penten-1-ol, (*E*)-2-pentenal-M, (*Z*)-2-methylpent-2-enal-M, (*Z*)-3-hexenol-M, (*E*,*E*)-2,4-hexadienal, (*E*)-2-hexenal-D, octanal-D, and (*Z*)-3-hexenol-D; the content of volatile organic compounds in FL was higher than that in BU, ST, and LE, including compounds such as 2-hydroxy-2-methyl-4-pentanone-M, 1-butanol,3-methyl, heptaldehyde-M, phenylacetaldehyde-M, benzaldehyde-M, 3-hydroxy-2-butanone-D, 2-hydroxy-2-methyl-4-pentanone-D, 1-octanol, nonanal-M, benzaldehyde-D, nonanal-D, phenylacetaldehyde-D, methylbenzoate-D, heptaldehyde-D, 1-octen-3-one,2-ethyl-1-hexa-nol, and ethylbenzene-D; these volatile organic compounds are not only important volatile organic compounds of Longya lilies but are also important flower flavor substances.

**Figure 5:**
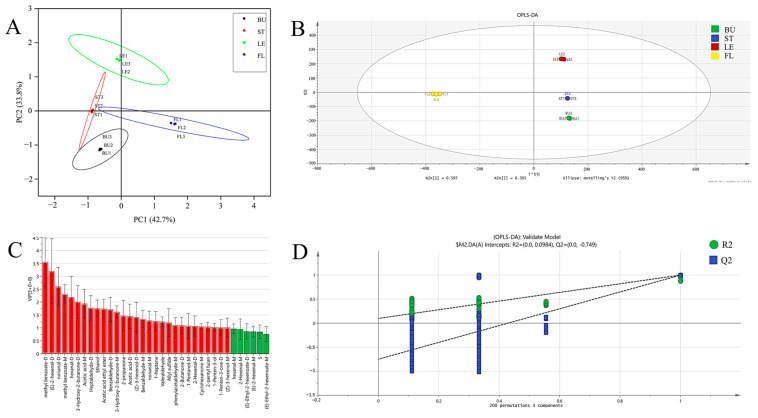
(**A**) PCA of different organs of Longya lily. (**B**) OPLS-DA. (**C**) OPLS-DA model’s variable projected importance (VIP) plot. (**D**) OPLS-DA model’s replacement test results.

In order to elucidate the differences among different organs of Longya lily, four samples of bulbs, stems, leaves, and flowers were subjected to PCA, and the results are shown in Figure 5A. PC1 and PC2 contributed 42.7% and 33.8%, respectively, bringing the total contribution to 76.5%, indicating that the first two major factor compounds could reflect the volatile organic compounds of Longya lilies more comprehensively. The results of principal component analysis showed that the differences in the volatile organic content of BU, ST, LE, and FL were all significant (*p* < 0.05), and the distribution of the characteristic components in the four organs of Longya lilies was characteristic and could be effectively differentiated. OPLS-DA is a supervised statistical analysis method for the visualization, discriminative analysis, and prediction of complex data [24]; it is used to determine the volatile organic compounds in the samples (Figure 5B), in which R^2^X was found to be 0.993, R^2^Y was found to be 0.998, and Q^2^ was found to be 0.996, indicating a good model fit. In addition, an alignment test with 200 cross-validations (Figure 5D) indicated that the OPLS-DA model was reliable; therefore, it could be used for the subsequent screening of differentially volatile organic compounds.

Variable importance in the projection (VIP) values is often used to identify key variables in OPLS-DA models [25], whereby a higher VIP value (VIP > 1 indicates a significant variable) indicates that the substance contributes more to the difference between samples. To further observe the differences, compounds with VIP values > 1 and *p* < 0.05 were screened as markers of differences using one-way ANOVA (Figure 5C), where methyl benzoate-D, nonanal-D, methyl benzoate-M, hexanal-D, 3-hydroxy-2-butanone-D, acetic acid-M, heptaldehyde-D, ethanol, acetic acid ethyl ester, benzaldehyde-D, 3-hydroxy-2-butanone-M, 2-propanone, acetic acid-D, (*Z*)-3-hexenol-D, benzaldehyde-M, nonanal-M, 1-heptene, valeraldehyde, allyl sulfide, and other VOCs were the key substances mainly affecting the flavor differences among the four organs of Longya lilies.

#### 2.4.2. Principal Component Analysis and Correlation Study of VOCs in Longya Lilies After Bud-Removal and Non-Bud-Removal Treatments

**Figure 6:**
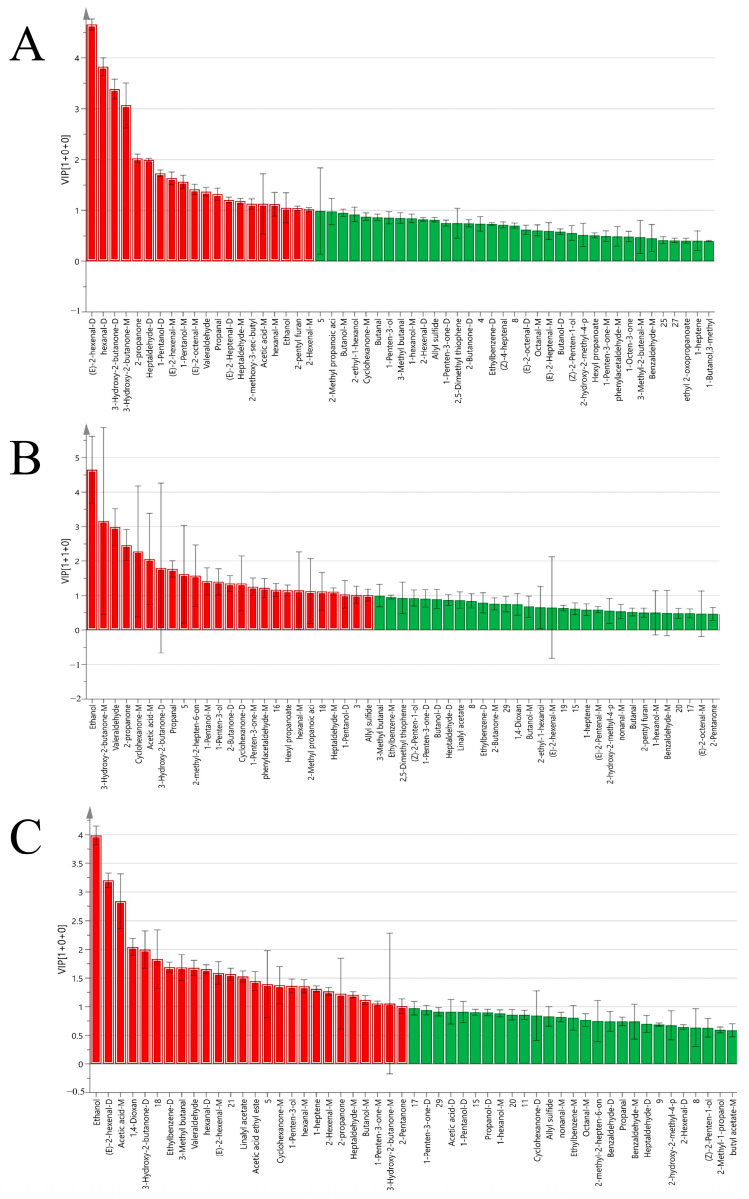
(**A**) VIP distributions of volatile organic compounds measured in bud-removal and non-bud-removal Longya lily bulbs, (**B**) stems, and (**C**) leaves, with red denoting labeled odor chemicals with VIP values > 1.0.

After visualizing 107 common volatile organic compounds in the bulbs, stems, and leaves of Longya lilies according to their corresponding fingerprints in both bud-removal and non-bud-removal treatments, compounds with VIP values > 1 and *p* < 0.05 were screened as markers of difference by one-way analysis of variance (ANOVA). The markers of difference are marked in red in Figure 6. Twenty odorants were measured from bud-removal and non-bud-removal bulbs, and the VOCs with the highest impact included two aldehydes and two ketones—(*E*)-2-hexenal-D, hexanal-D, 3-hydroxy-2-butanone-D, and 3-hydroxy-2-butanone-M (Figure 6A). A total of 22 odorants were measured from bud-removal and non-bud-removal stems, with the most influential VOCs being ethanol (Figure 6B). In total, 25 odorants were measured from bud-removal and non-bud-removal leaves (VIP > 1 and *p* < 0.05), with ethanol, (*E*)-2-hexenal-D, and acetic acid-M being the main discriminators (Figure 6C).

**Figure 7:**
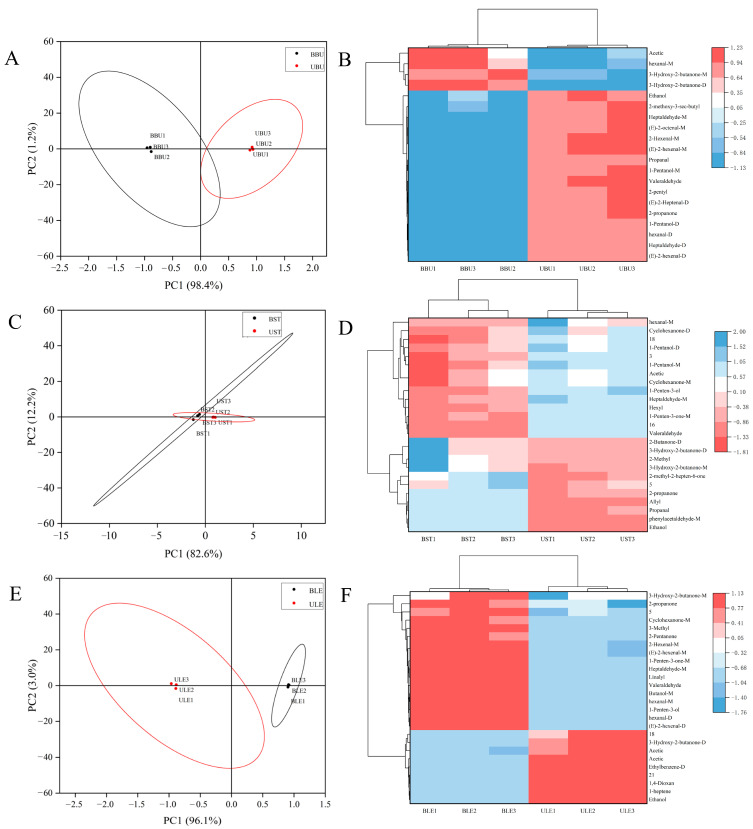
PCA and cluster heatmap of marker volatiles based on VIP values acquired from bud-removal and non-bud-removal treatments of Longya lily (**A**,**B**) bulbs, (**C**,**D**) stems, and (**E**,**F**) leaves.

In this study, the screened differential markers were also analyzed using PCA and a clustering heat map to differentiate between bud-removal and non-bud-removal Longya lily samples (Figure 7). The PCA scoring plots of the 20 labeled compounds (Figure 7A) could effectively differentiate between bud-removal and non-bud-removal bulbs, with a total contribution of 99.6% for PC1 (98.4%) and PC2 (1.2%), and the clustering heat maps further illustrated that the bud-removal bulb levels of acetic acid-M, hexanal-M, 3-hydroxy-2-butanone-D, and 3-hydroxy-2-butanone-M were higher than that of the non-bud-removal bulbs (Figure 7B); similarly, the levels of aldehydes, alcohols, and heterocyclic compounds ethanol, 2-methoxy-3-sec-butyl pyrazine, heptaldehyde-M, (*E*)-2-octenal-M, (*E*)-2-hexenal-M, propanal, 1-pentanol-M, and valeraldehyde were higher than that in bud-removal bulbs. In contrast, the aggregation contribution of PC1 (82.6%) and PC2 (12.2%) in the bud-removal and non-bud-removal Longya lily stems was 94.8% (Figure 7C), with high aggregation among samples and some areas of overlap, and the separation of the characteristic substances was not obvious (Figure 7D). The PCA scoring plot (Figure 7E) could also effectively differentiate between bud-removal and non-bud-removal leaves, and its differential markers are also shown in the clustering heat map. There is a favorable classification effect of odor characteristics in bud-removal and non-bud-removal leaves (Figure 7F).

#### 2.4.3. Volatile Organic Compounds

**Figure 8:**
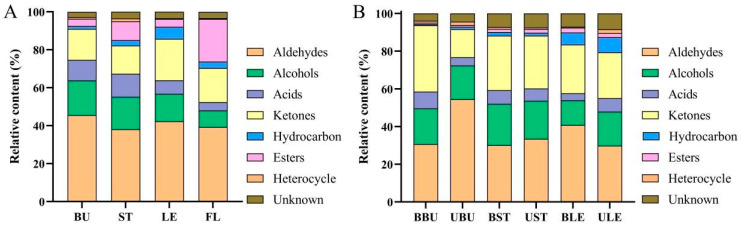
Pile-up bar graphs of volatile organic compounds in four organs of Longya lilies. (**A**) Bud removed. (**B**) Non-bud-removed Longya lilies.

To visually analyze the contents of various types of VOCs in Longya lilies, the relative contents of VOCs in Longya lily were obtained by conversion using the normalization method based on the ionic peak volumes of different flavor compounds, as shown in Table 2. The stacking diagrams were plotted based on the relative contents of various types of compounds (Figure 8). As shown in the figure, the VOCs identified in the bulbs mainly included aldehydes (45.58%) and alcohols (18.26%), with the highest contents of hexanal-D (17.94%) and ethanol (8.71%), which were consistent with previous studies [26,27]. Hexanal-D can give the bulbs of Longya lilies a fresh, herbaceous aroma. It can be used in the preparation of flavors and also has the effect of preventing fruit deterioration, and it can be used as a plasticizer [28]. Among the four Longya lily organs, FL had the highest total VOC content, followed by LE, while ST and BU had the lowest content. Alcohols, ketones, alkanes, and esters were the main sources of the VOCs of lilies [29,30], and the relative content of ester in FL was 5.99, 2.26, and 5.39 times that of BU, ST, and LE, respectively (Figure 8A). The results showed that the volatile organic compounds of Longya lily BU, ST, LE, and FL were complex and contained a variety of chemical elements; the relative contents of flavor substances were mainly composed of aldehydes (38.09–45.58%), ketones (14.85–21.86%), alcohols (8.74–18.26%), and esters (3.76–22.53%).

However, the distribution of volatiles in bud-removal and non-bud-removal Longya lilies exhibited significant differences (Figure 8B). It is worth noting that the total VOC content in the bulbs of the non-bud-removal treatment was considerably higher than that in the bud-removal bulbs. The percentage of ketones in the bud-removal bulbs (BBU) surpassed that in the non-bud-removal bulbs (UBU), with 3-hydroxy-2-butanone-M constituting 13.29%,which was the primary contributor to the overall aroma, followed by 3-hydroxy-2-butanone-D at 12.05%. In contrast, the aldehyde percentage was higher in the UBUs, with hexanal-D accounting for 16.53%, followed by (E)-2-hexenal-D (13.89%). Although the total amount of VOCs in the stems (ST) and leaves (LE) was greater in the bud-removal treatment compared to the non-bud-removal treatment, the difference was not statistically significant. Aldehydes (29.86–54.51%), ketones (14.92–35.16%), and alcohols (13.09–21.92%) were identified as the primary odorants in the bulbs, stems, and leaves of Longya lilies under both crop management methods.

## 3. Materials and Methods

### 3.1. Sample Preparation and Collection

**Figure 9:**
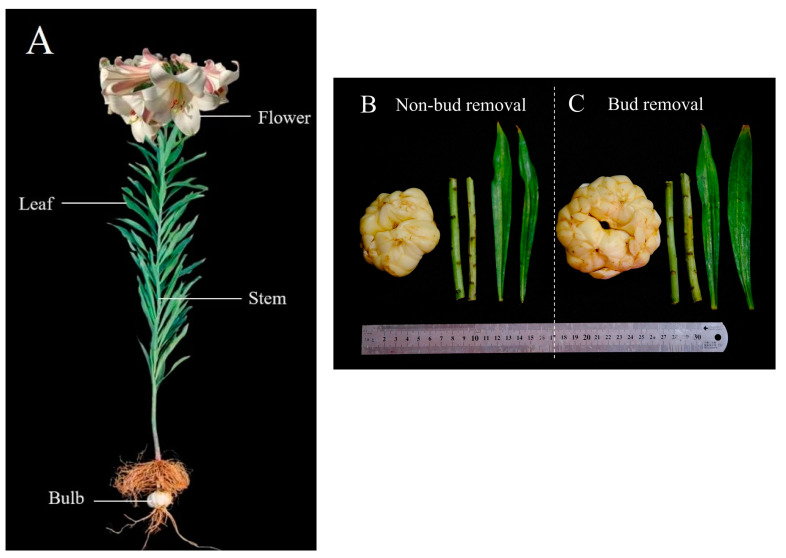
Appearance of different organs and planting methods of Longya lily. (**A**) Longya lily bulbs, stems, leaves, and flowers; (**B**) bulbs, stems, and leaves of the non-bud-removal treatment; (**C**) bulbs, stems, and leaves of the bud-removal treatment.

In this study, Longya lily was selected from Longhui County, Shaoyang City, Hunan Province, China. A two-factor and two-level factorial design was used to set up a plot of 9 m long and 10 m wide for bud-removal and non-bud-removal Longya lilies. The plot was sown by a trench spacing of 20 cm, a sowing depth of 1 cm, and a strip width of 10 cm. Fertilization and field management were carried out in a unified manner. Bud-removal treatment was used as a control when the buds grew to about 5 cm each year, and the effect on the plants was evaluated by two treatment methods. The bulbs, stems, leaves, and flowers of the Longya lily (Figure 9A) were collected in May 2024 and named BU, ST, LE, and FL, respectively. The bulbs, stems, and leaves of Longya lilies under the growth of the non-bud-removal treatment and the bud-removal treatment were harvested at maturity in August 2024; the treatments were named UBU, UST, and ULE, as well as BBU, BST, and BLE, respectively (Figure 1B). After they were separated and washed with water in the laboratory, they were pre-frozen at −80 °C for 12 h, then vacuum freeze-dried at a cold trap temperature of −40 °C and a vacuum of 50 Pa for 36 h. After drying, they were pulverized, sieved (80 mesh), and stored immediately in a sealed bag in a cool, dry room at −40 °C for later use.

### 3.2. Chemicals and Instruments

Test reagents: 2-butanone (CAS registry No. 78-93-3; purity ≥ 98%), 2-pentanone (CAS registry No. 107-87-9; purity ≥ 98%), 2-hexanone (CAS registry No. 591-78-6; purity ≥ 98%), 2-Heptanone (CAS registry No. 110-43-0; purity ≥ 98%), 2-octanone (CAS registry No. 111-13-7; purity ≥ 98%), and 2-nonanone (CAS registry No. 821-55-6; purity ≥ 98%) were purchased from Aladdin Company (Shanghai, China).

Instruments: 1/10,000 balance (Model BSA 224S-CW, Sartorius Scientific Instruments Ltd., Beijing, China); vacuum freeze-dryer (Model SCIENTZ-10N/A, Ningbo scientz biotechnology Co., Ltd., Ningbo, China); and high-speed multi-functional grinder (Model TQ-500Y, Yongkang Tianqi Shengshi Industry and Trade Co., Ltd., Yongkang, China).

### 3.3. GC-IMS Analysis

VOCs were detected in all samples using a GC-IMS system (FlavourSpec^®^, Gesellschaft für Analytische Sensorsysteme mbH, Dortmund, Germany) and an autosampler (CTC Analytics AG, Zwingen, Switzerland). For the experiment, three samples were weighed from each batch. The samples were accurately weighed (0.5 g) into a 20 mL headspace bottle (Shandong Haineng Scientific Instruments, Dezhou China). The parameters of the instrument were set as follows:

GC-IMS conditions—incubation temperature: 80 °C; incubation time: 15 min; incubation speed: 500 r/min; injection needle temperature: 110 °C. Headspace injection was used, whereby the injection temperature was 60 °C, the injection volume was 200 µL, and the carrier gas was high-purity nitrogen (purity ≥ 99.999%). The chromatographic column was an MXT-WAX capillary column (15 m × 0.53 mm, 1.0 μm, Beijing, China). The flow rate of the program was as follows: the initial flow rate of 2.0 mL/min was kept for 2 min, linearly increased to 10.0 mL/min for 8 min, linearly increased to 100.0 mL/min for 10 min, and then kept for 20 min at the end of the program; the length of the migration tube was 53 mm, the temperature was 45 °C, the electric field strength was 500 V/cm, the drift gas was high-purity nitrogen (purity ≥ 99.999%), and the gas flow rate of the drift tube was 150.0 mL/min.

The mixed standards of the six ketones (2-butanone, 2-pentanone, 2-hexanone, 2-Heptanone, 2-octanone, and 2-nonanone) were detected, and the calibration curves of retention time and retention index were established. Subsequently, the retention index of the substance was calculated from the retention time of the target, and the target was characterized using the built-in GC retention index database of the VOCal (0.4.10) software (NIST 2020) and the IMS migration time database for searching and comparing. The relative content of each VOC in Longya lilies was calculated by normalizing the area of each chromatographic peak to the ratio of the total peak area.

### 3.4. Statistical Analysis

Reporter, Gallery Plot, and other plug-ins in VOCal data processing software were used to generate 3D spectra, 2D spectra, difference spectra, and fingerprints of volatile constituents for the comparison of VOCs between samples. Origin 2022 software (OriginLab Co., Northampton, MA, USA) was used for PCA and thermogram analysis. OPLS-DA was run using SIMCA-P 14.1 software (Umerics, Umea, Sweden), and relative content stacked plots were generated using GraphPad Prism 9.5.0.

## 4. Conclusions

As a plant that combines medicinal, edible, and ornamental values, the unique versatility of lilies enriches people’s daily lives and serves as a significant driver of economic growth in several regions. In this study, a comprehensive and comparative volatile analysis method was employed to characterize and differentiate Longya lily from various organs and different crop management methods. Overall, 93 VOCs were identified across seven compound classes, with aldehydes, alcohols, ketones, and esters contributing most significantly to the Longya lily. Combining GC-IMS spectra, PCA, and heatmap clustering analysis, it was found that there are both differences and similarities among the VOCs of various organs. The differences are manifested in the varying levels of the same VOCs across different organs [31,32,33]. Among the four organs, the total VOC content in the flowers is the highest, with the main components being alcohols, esters, and phenols, which is consistent with previous research findings. Studies have shown that the IC50 values of the essential oil extracted from lily flowers and vitamin C (VC) against DPPH**·** are 0.31 mg/mL and 0.36 mg/mL, respectively, demonstrating potent antioxidant effects [34,35]. It can also directly stimulate the secretion of the pituitary gland, enzymes, and hormones, balancing bodily functions and serving a beautifying and maintenance role [36]. Therefore, the application of lily flower extract as a supplement in cosmetic formulations is quite promising. Compared to the bud-removal treatment, the abundance of VOCs in the non-bud-removed bulbs of the Longya lily significantly increased, but the total VOC content in the stems and leaves under both treatments did not show significant differences. Notably, in the bulbs without bud-removal treatment, compounds such as ethanol, 2-methoxy-3-sec-butyl pyrazine, heptaldehyde-M, (E)-2-octenal-M, and (E)-2-hexenal-M have been identified as volatile organic compounds. Therefore, if volatile oil is to be developed as the main raw material, the optimal crop management method should be the non-bud-removal treatment, as this results in the highest VOC content in the Longya lily bulbs. Despite these findings, we recognize that our research has some limitations. Because our study focused only on the characterization of VOCs from four organs of the Longya lily—bulbs, stems, leaves, and flowers—there may be some bias in the results. Our future research will include a wider range of lily varieties, lily tissue types such as fibrous roots and bead buds, and different crop management methods for lilies. This study can provide a theoretical basis for the rational utilization of different organs of Longya lily, provide a theoretical basis for studying the flavor differences of Longya lily under different crop management methods, and finally, aid in determining the best method.

## Figures and Tables

**Table 1:** The characteristic aroma components of Longya lily from four different organs, as well as those subjected to bud-removal and non-bud-removal treatments.

Count	Compound	CAS#	Formula	MW	RI	Rt [sec]	Dt [a.u.]	Flavor Description
1	**1**	unidentified	*	0	1169.9	376.591	1.19667	/
2	**2**	unidentified	*	0	1460.6	862.462	1.20404	/
3	methyl benzoate-D	C93583	C_8_H_8_O_2_	136.1	1583.5	1162.145	1.59803	wintergreen, almond, floral, cananga
4	methyl benzoate-M	C93583	C_8_H_8_O_2_	136.1	1583	1160.532	1.22269	wintergreen, almond, floral, cananga
5	Acetic acid-D	C64197	C_2_H_4_O_2_	60.1	1466.7	875.513	1.16392	spicy
6	Acetic acid-M	C64197	C_2_H_4_O_2_	60.1	1463.9	869.582	1.05376	spicy
7	Benzaldehyde-M	C100527	C_7_H_6_O	106.1	1495.9	939.747	1.14947	bitter almond, cherry, nutty
8	Benzaldehyde-D	C100527	C_7_H_6_O	106.1	1495.9	939.747	1.46835	bitter almond, cherry, nutty
9	1-Octanol	C111875	C_8_H_18_O	130.2	1554.4	1083.003	1.47033	citrus, sweet, herbs, waxy, rose, mushroom
10	Propanoic acid	C79094	C_3_H_6_O_2_	74.1	1543.5	1054.513	1.10979	yogurt, vinegar
11	phenylacetaldehyde-M	C122781	C_8_H_8_O	120.2	1610.2	1239.896	1.25793	hyacinth, sweet fruity, almond, cherry, clover honey, cocoa
12	phenylacetaldehyde-D	C122781	C_8_H_8_O	120.2	1608	1233.039	1.53866	hyacinth, sweet fruity, almond, cherry, clover honey, cocoa
13	(*E,E*)-2,4-heptadienal	C4313035	C_7_H_10_O	110.2	1477.8	899.391	1.19427	fatty, oily, aldehyde, vegetable, cinnamon
14	(*Z*)-3-hexenol-D	C928961	C_6_H_12_O	100.2	1397.7	740.494	1.50195	green, herb
15	(*Z*)-3-hexenol-M	C928961	C_6_H_12_O	100.2	1399.3	743.433	1.2367	green, herb
16	(*E*)-Ethyl-2-hexenoate-D	C27829727	C_8_H_14_O_2_	142.2	1336.6	638.609	1.80277	fruity, vegetable
17	(*E*)-Ethyl-2-hexenoate-M	C27829727	C_8_H_14_O_2_	142.2	1337.1	639.327	1.33034	fruity, vegetable
18	(*Z*)-2-Penten-1-ol	C1576950	C_5_H_10_O	86.1	1345.6	652.643	0.94494	green, plastic, rubber
19	1-Penten-3-ol	C616251	C_5_H_10_O	86.1	1175.1	383.645	0.94119	ethereal, green, tropical fruity
20	(*E*)-2-Heptenal-D	C18829555	C_7_H_12_O	112.2	1340.7	644.937	1.66537	spicy, green vegetables, fresh, fatty
21	nonanal-M	C124196	C_9_H_18_O	142.2	1396.2	737.808	1.47457	rose, citrus, strong oily
22	nonanal-D	C124196	C_9_H_18_O	142.2	1402.1	748.498	1.9419	rose, citrus, strong oily
23	**3**	unidentified	*	0	1425.4	791.89	1.44527	/
24	**4**	unidentified	*	0	1411.7	766.005	1.26022	/
25	1-hexanol-M	C111273	C_6_H_14_O	102.2	1373.7	698.59	1.32522	fresh, fruity, wine, sweet, green
26	1-hexanol-D	C111273	C_6_H_14_O	102.2	1373.4	698.21	1.64602	fresh, fruity, wine, sweet, green
27	**5**	unidentified	*	0	1386.2	720.223	1.10028	/
28	2-methyl-2-hepten-6-one	C110930	C_8_H_14_O	126.2	1354.9	667.545	1.17293	citrus, fruity, moldy, ketone
29	3-Hydroxy-2-butanone-D	C513860	C_4_H_8_O_2_	88.1	1292.8	572.967	1.32387	butter, cream
30	3-Hydroxy-2-butanone-M	C513860	C_4_H_8_O_2_	88.1	1291.9	571.182	1.06837	butter, cream
31	1-Octen-3-one	C4312996	C_8_H_14_O	126.2	1318.1	610.458	1.27406	strong earthy, mushroom, vegetable, fishy, chicken
32	Cyclohexanone-M	C108941	C_6_H_10_O	98.1	1292.8	572.967	1.15354	strong, pungent, earthy
33	Cyclohexanone-D	C108941	C_6_H_10_O	98.1	1294.6	576.538	1.45082	strong, pungent, earthy
34	**6**	unidentified	*	0	1300.2	584.571	1.63401	/
35	1-Pentanol-D	C71410	C_5_H_12_O	88.1	1260.8	514.052	1.51188	balsamic
36	1-Pentanol-M	C71410	C_5_H_12_O	88.1	1261.3	514.945	1.25317	balsamic
37	(*E*)-2-hexenal-D	C6728263	C_6_H_10_O	98.1	1227.5	459.155	1.51349	green, banana, fat
38	(*E*)-2-hexenal-M	C6728263	C_6_H_10_O	98.1	1229.3	462.074	1.17803	green, banana, fat
39	2-pentyl furan	C3777693	C_9_H_14_O	138.2	1239.5	478.302	1.2495	bean, fruity, earthy, green, vegetable
40	Heptaldehyde-D	C111717	C_7_H_14_O	114.2	1195.1	411.362	1.69333	fresh, aldehyde, fatty, green herbs, wine, fruity
41	Heptaldehyde-M	C111717	C_7_H_14_O	114.2	1197	414.067	1.33789	resh, aldehyde, fatty, green herbs, wine, fruity
42	Ethylbenzene-D	C100414	C_8_H_10_	106.2	1145.6	345.742	1.44536	aromatic odor
43	Ethylbenzene-M	C100414	C_8_H_10_	106.2	1141.4	340.675	1.07501	aromatic odor
44	Allyl sulfide	C592881	C_6_H_10_S	114.2	1138.3	336.989	1.33025	garlic
45	hexanal-D	C66251	C_6_H_12_O	100.2	1097.7	292.071	1.54566	fresh, green, fat, fruity
46	hexanal-M	C66251	C_6_H_12_O	100.2	1092.6	287.381	1.27156	fresh, green, fat, fruity
47	1,4-Dioxan	C123911	C_4_H_8_O_2_	88.1	1081.9	278.003	1.13124	pungent, ether
48	(*Z*)-4-heptenal	C6728310	C_7_H_12_O	112.2	1252.8	500.362	1.1452	grass, oil
49	2-Hexenal-D	C505577	C_6_H_10_O	98.1	1213.3	437.675	1.50904	sweet almonds, fruity, green leaves, apples, plums, vegetables
50	2-Hexenal-M	C505577	C_6_H_10_O	98.1	1213.7	438.253	1.18088	sweet almonds, fruity, green leaves, apples, plums, vegetables
51	(*Z*)-2-Methylpent-2-enal-D	C623369	C_6_H_10_O	98.1	1161.8	366.033	1.48549	aldehydes, soil, garlic, ripe cherries
52	(*Z*)-2-Methylpent-2-enal-M	C623369	C_6_H_10_O	98.1	1161.8	366.033	1.15671	aldehydes, soil, garlic, ripe cherries,
53	4-Heptanone-D	C123193	C_7_H_14_O	114.2	1170.2	377.008	1.57425	fruity
54	4-Heptanone-M	C123193	C_7_H_14_O	114.2	1170.2	377.008	1.22172	fruity
55	Butanol-M	C71363	C_4_H_10_O	74.1	1154.7	357.01	1.17922	wine
56	(*E*)-2-Pentenal-M	C1576870	C_5_H_8_O	84.1	1124.3	320.671	1.10921	potato, peas
57	(*E*)-2-Pentenal-D	C1576870	C_5_H_8_O	84.1	1122.5	318.72	1.36298	potato, peas
58	2-hexanone-M	C591786	C_6_H_12_O	100.2	1116.8	312.379	1.19797	fruity, fungal, meaty, buttery
59	2-hexanone-D	C591786	C_6_H_12_O	100.2	1117	312.623	1.503	fruity, fungal, meaty, buttery
60	**7**	unidentified	*	0	1111.5	306.526	1.08806	/
61	butyl acetate-M	C123864	C_6_H_12_O_2_	116.2	1086.4	281.893	1.2377	fruity
62	butyl acetate-D	C123864	C_6_H_12_O_2_	116.2	1084.5	280.186	1.61745	fruity
63	2,5-Dimethyl thiophene	C638028	C_6_H_8_S	112.2	1187.1	400.177	1.07926	nutty, sulfury
64	**8**	unidentified	*	0	1061.4	260.8	1.19748	/
65	Propanol-M	C71238	C_3_H_8_O	60.1	1049.9	251.667	1.11275	alcohol, pungent
66	Propanol-D	C71238	C_3_H_8_O	60.1	1049.2	251.141	1.25578	alcohol, pungent
67	1-Penten-3-one-D	C1629589	C_5_H_8_O	84.1	1038.8	243.136	1.3087	strong, pungent odors
68	1-Penten-3-one-M	C1629589	C_5_H_8_O	84.1	1040.6	244.541	1.0782	strong, pungent odors
69	Ethanol	C64175	C_2_H_6_O	46.1	937.8	182.624	1.11856	aromaticity
70	3-Methyl butanal	C590863	C_5_H_10_O	86.1	931.5	179.591	1.39948	chocolate, fat
71	2-Pentanone	C107879	C_5_H_10_O	86.1	1020.5	229.743	1.38929	acetone, fresh, sweet fruity, wine
72	2,3-Butanedione	C431038	C_4_H_6_O_2_	86.1	991.4	210.382	1.17293	butter, popcorn, sweet taste, sour rice
73	Valeraldehyde	C110623	C_5_H_10_O	86.1	1000.6	215.981	1.41874	green grassy, faint banana, pungent
74	**9**	unidentified	*	0	1050.8	252.37	0.94639	/
75	**10**	unidentified	*	0	1012.2	223.912	1.08232	/
76	Propanal	C123386	C_3_H_6_O	58.1	815.5	132.229	1.13813	pungent, green grassy
77	2-propanone	C67641	C_3_H_6_O	58.1	837.9	140.273	1.11396	fresh, apple, pear
78	**11**	unidentified	*	0	763.6	115.309	1.13289	/
79	**12**	unidentified	*	0	712.2	100.659	1.14176	/
80	1-heptene	C592767	C_7_H_14_	98.2	786.5	122.467	1.08648	gasoline
81	**13**	unidentified	*	0	792.8	124.54	1.1205	/
82	2-Methyl propanal	C78842	C_4_H_8_O	72.1	830.7	137.617	1.27928	banana, melon, slightly nutty
83	**14**	unidentified	*	0	853.6	146.206	1.15295	/
84	Butanal	C123728	C_4_H_8_O	72.1	893.8	162.566	1.27842	pungent, fruity, green leaf
85	Acetic acid ethyl ester	C141786	C_4_H_8_O_2_	88.1	900.1	165.289	1.33457	fresh, fruity, sweet, grassy
86	**15**	unidentified	*	0	798	126.256	0.96012	/
87	2-Butanone-D	C78933	C_4_H_8_O	72.1	917.5	173.073	1.24453	fruity, camphor
88	2-Butanone-M	C78933	C_4_H_8_O	72.1	916.7	172.686	1.0679	fruity, camphor
89	**16**	unidentified	*	0	868.1	151.895	1.05772	/
90	3-Methyl-2-butenal-M	C107868	C_5_H_8_O	84.1	1212	435.627	1.09106	fruity
91	3-Methyl-2-butenal-D	C107868	C_5_H_8_O	84.1	1210.7	433.784	1.35477	fruity
92	4-Methyl-2-pentanone	C108101	C_6_H_12_O	100.2	1023.5	231.888	1.47906	ketone
93	2-hydroxy-2-methyl-4-pentanone-M	C123422	C_6_H_12_O_2_	116.2	1372.5	696.599	1.13723	mild, pleasant
94	2-hydroxy-2-methyl-4-pentanone-D	C123422	C_6_H_12_O_2_	116.2	1372.4	696.508	1.52947	mild, pleasant
95	Hexyl propanoate	C2445763	C_9_H_18_O_2_	158.2	1349.2	658.425	1.42698	sweet fruity, earthy, pungent aroma resembling rotten fruits
96	ethyl 2-oxopropanoate	C617356	C_5_H_8_O_3_	116.1	1277.1	543.21	1.15559	fruity, sweet rum, vegetable caramel
97	Octanal-M	C124130	C_8_H_16_O	128.2	1303.7	589.545	1.41017	aldehyde, waxy, citrus, orange, fruity
98	Octanal-D	C124130	C_8_H_16_O	128.2	1303.4	589.119	1.81914	aldehyde, waxy, citrus, orange, fruity
99	**17**	unidentified	*	0	1195.6	412.137	1.43025	/
100	**18**	unidentified	*	0	1320.8	614.48	1.15761	/
101	**19**	unidentified	*	0	996.8	213.472	1.10373	/
102	**20**	unidentified	*	0	1112.5	307.631	1.34941	/
103	**21**	unidentified	*	0	1098.2	292.531	1.36487	/
104	**22**	unidentified	*	0	997.2	213.687	1.46595	/
105	2-Methyl-1-propanol	C78831	C_4_H_10_O	74.1	1104.5	299.062	1.17395	fresh, alcoholic, leather
106	**23**	unidentified	*	0	1107.3	302.095	1.10657	/
107	**24**	unidentified	*	0	1067	265.431	1.80481	/
108	(*E*)-2-octenal-D	C2548870	C_8_H_14_O	126.2	1427.1	795.165	1.81291	fresh cucumber, fatty, green herbal, banana, green leaf
109	(*E*)-2-octenal-M	C2548870	C_8_H_14_O	126.2	1426.7	794.437	1.33114	fresh cucumber, fatty, green herbal, banana, green leaf
110	**25**	unidentified	*	0	1410.5	763.929	1.77584	/
111	**26**	unidentified	*	0	1357.7	671.983	1.10533	/
	2-methoxy-3-sec-butyl							moldy, green, vegetable, nutty,
112	pyrazine	C24168705	C_9_H_14_N_2_O	166.2	1509.3	970.767	1.26067	pepper, potato, fishy, galbanum
113	2-ethyl-1-hexanol	C104767	C_8_H_18_O	130.2	1513.4	980.45	1.40846	citrus, fresh floral, greasy
114	**27**	unidentified	*	0	1436.3	813.124	1.1974	/
115	2-Methyl propanoic acid	C79312	C_4_H_8_O_2_	88.1	1576.7	1143.045	1.15493	yogurt, rancid cream
116	(*E*,*E*)-2,4-hexadienal	C142836	C_6_H_8_O	96.1	1408.6	760.381	1.1187	sweet, green, floral, citrus
117	**28**	unidentified	*	0	1252.9	500.488	1.20531	/
118	(*E*)-2-Heptenal-M	C18829555	C_7_H_12_O	112.2	1340.8	645.07	1.25453	spicy, green vegetables, fresh, fatty
119	Butanol-D	C71363	C_4_H_10_O	74.1	1153.9	356.001	1.38279	wine
120	**29**	unidentified	*	0	1146.7	347.035	1.11051	/
121	1-Butanol, 3-methyl	C123513	C_5_H_12_O	88.1	1217.5	443.959	1.24245	whiskey, banana, fruity
122	**30**	unidentified	*	0	897.1	164.007	1.0438	/
123	**31**	unidentified	*	0	1083.1	279	1.51066	/
124	Linalyl acetate	C115957	C_12_H_20_O_2_	196.3	1547.5	1064.871	1.21531	lily of the valley, lavender

Note: “*”: did not find Formula; “/”:was not flavor description; The substance suffixes M and D are the monomer and dimer of the same substance, respectively, and the number repr-esents the unidentified peak; the odor descriptions of the above volatile organic compounds are sourced from the website http://www.flavornet.org, accessed on 13 September 2024.

**Table 2:** The relative volatile organic compounds of Longya lily from four different organs, as well as those subjected to bud-removal and non-bud-removal treatments.

Sample	Aldehydes	Alcohols	Acids	Ketones	Hydrocarbon	Esters	Heterocycle	Unknown
BU	45.58 ± 0.30 d	18.26 ± 0.30 d	10.84 ± 0.91 c	16.33 ± 0.25 b	1.56 ± 0.05 a	3.76 ± 0.23 a	0.86 ± 0.03 b	2.80 ± 0.14 a
ST	38.09 ± 0.10 a	17.12 ± 0.05 c	12.16 ± 0.26 c	14.85 ± 0.08 a	2.83 ± 0.07 b	9.98 ± 0.11 c	1.47 ± 0.05 c	3.50 ± 0.03 b
LE	42.29 ± 0.12 c	14.50 ± 0.12 b	7.10 ± 0.78 b	21.86 ± 0.52 d	6.33 ± 0.07 d	4.18 ± 0.11 b	0.34 ± 0.03 a	3.40 ± 0.12 b
FL	39.28 ± 0.19 b	8.74 ± 0.62 a	4.35 ± 0.32 a	18.01 ± 0.19 c	3.35 ± 0.03 c	22.53 ± 0.12 d	0.38 ± 0.01 a	3.35 ± 0.16 b
BBU	30.68 ± 0.27 a	18.98 ± 0.12 c	8.88 ± 0.52 c	35.16 ± 0.33 e	0.62 ± 0.03 a	0.76 ± 0.04 a	1.02 ± 0.19 b	3.90 ± 0.19 a
BST	54.51 ± 0.49 d	17.87 ± 0.14 b	4.40 ± 0.31 a	14.92 ± 0.55 a	1.25 ± 0.04 b	0.81 ± 0.06 a	1.83 ± 0.07 c	4.42 ± 0.45 a
BLE	30.13 ± 0.32 a	21.92 ± 0.34 e	7.20 ± 0.27 b	28.96 ± 0.92 d	1.91 ± 0.04 d	1.73 ± 0.02 b	0.96 ± 0.08 b	7.19 ± 0.50 b
UBU	33.45 ± 0.81 b	20.20 ± 0.21 d	6.55 ± 0.60 b	28.01 ± 0.16 d	1.49 ± 0.03 c	2.02 ± 0.01 c	0.80 ± 0.02 b	7.48 ± 0.20 b
UST	40.84 ± 0.26 c	13.09 ± 0.18 a	3.66 ± 0.33 a	25.89 ± 0.26 c	6.38 ± 0.13 e	2.55 ± 0.02 e	0.49 ± 0.08 a	7.10 ± 0.15 b
ULE	29.86 ± 0.59 a	18.04 ± 0.31 b	7.16 ± 0.35 b	24.23 ± 0.48 b	8.04 ± 0.11 f	2.22 ± 0.12 d	2.01 ± 0.08 c	8.44 ± 0.35 c

Note: Data are presented as the mean ± standard deviation of three replicates. Different letters in the same line indicate a significant difference (*p* < 0.05).

## Data Availability

Data are contained within the article and Supplementary Materials.

## Supplementary Materials

The following supporting information can be downloaded at: https://www.mdpi.com/article/10.3390/molecules30061238/s1. Table S1. Identification of Volatile Organic Compounds in Longya Lily; Table S2. Relative contents of VOCs in different organs of Longya Lilies; Table S3. Relative contents of VOCs in Longya Lilies Undergoing Bud-Removal and Non-Bud-Removal Treatments.

## Abbreviations

The following abbreviations are used in this manuscript:

GC-IMS	Gas chromatography–ion mobility spectrometry
VOCs	Volatile organic compounds
E-nose	Electronic nose
GC-O	Gas chromatography–olfactometry
OPLS-DA	Partial least squares discriminant analysis
VIP	Variable importance
